# O09 Isolated muscular sarcoidosis revealed by calcitriol-mediated hypercalcaemia and fluorodeoxyglucose positron emission tomography

**DOI:** 10.1093/rap/rkaa053.008

**Published:** 2020-11-03

**Authors:** Constanta Amoasii, Ines Soares, Claire Dubois

**Affiliations:** Liverpool University Hospitals NHS Foundation Trust, Liverpool, United Kingdom

## Abstract

**Case report - Introduction:**

Sarcoidosis, a multisystem disorder of unknown aetiology, commonly presents with bilateral hilar adenopathy, pulmonary infiltrates, skin and/or eye lesions. Musculoskeletal and neuromuscular manifestations occur less commonly.

Calcitriol-mediated hypercalcaemia is a known complication of sarcoidosis. Severe hypercalcaemia, however, is detected in ˂5% of patients and pseudo-malignant hypercalcaemia (>3.5 mmol/L) is very unusual.

There are limited reports of isolated granulomatous myositis causing hypercalcaemia. Our patient uniquely presented with symptoms of hypercalcaemia and PET-scan incidentally revealed sarcoid-like myositis. This case reports an unusual presentation of life-threatening hypercalcaemia revealing sarcoid myositis that rheumatologists and physicians should be aware of.

**Case report - Case description:**

The patient, a 69-year old Caucasian female, was referred to Rheumatology after a PET-scan identified features of sarcoid myositis. She was initially admitted due to hypercalcaemia and 2 months of tiredness, lethargy, generalised weakness, and nausea. She had difficulty in climbing stairs but was independently mobile with no difficulty standing from sitting. History included biopsy-proven cutaneous lupus (on Hydroxychloroquine, which she stopped weeks prior to admission), chronic interstitial cystitis and CKD stage 3A without proteinuria.

Clinical examination was unremarkable. Bloods showed hypercalcaemia (3.62 mmol/L) with inappropriately normal PTH (1.8 qmol/L) and a raised ACE (72 U/L). PET-scan showed symmetric, diffuse, and heterogeneous muscular activity within the posterior thighs, anterior to the hips and psoas muscles, reported as possible sarcoid-like myositis.

PET-scan findings were discussed with nuclear medicine team and deemed non-specific. Remaining results included phosphate 1.28, ALP 78, vitamin D3 64, urea 8.6, creatinine 97, e-GFR 49, ESR 10 and CK 30. Normal CXR, CT head and MIBI NM parathyroid scan. CT thorax, abdomen and pelvis showed bladder calcification.

Calcium level and symptoms resolved with IV fluid, IV pamidronate and zoledronic acid. She was discharged with follow up arranged.

Follow-up bloods revealed high 1,25 vitamin D of 259 (43-144pmol/L) raising the possibility of calcitriol-mediated hypercalcaemia from granulomatous myositis.

EMG showed borderline myopathic results. Calcium levels and symptoms remained stable, so no treatment was initiated until 6 months following initial presentation, when she was readmitted with severe hypercalcaemia, similar symptomatology, and normal examination. MRI thighs showed changes consistent with myositis and muscle biopsy revealed granulomatous inflammation consistent with sarcoid. Myositis autoantibodies (anti Ro-52) came back positive.

After 10 months of multiple investigations and monitoring, a diagnosis of sarcoid myositis has been made and patient initiated on a Prednisolone tapering regimen; her response is yet to be assessed.

**Case report - Discussion:**

In patients with granulomatous disease, such as sarcoidosis, hypercalcaemia occurs because of ectopic 25(OH)D-1-hydroxylase expression in macrophages and formation of excessive amounts of 1,25(OH)2D. In a review of 101 adult cases of calcitriol-mediated hypercalcaemia, 48% were eventually diagnosed with sarcoidosis.

Sarcoid myositis, as described by Aubart et all, has four patterns: nodular; smouldering phenotype; acute, subacute, or progressive myopathic; and combined myopathic/neurogenic type. In all patterns, sarcoidosis was multi-visceral with a median of 3 extra muscular organs involved (mostly lungs, lymph nodes, eyes, and skin). Our patient, however, did not fit into one of these patterns given her single-organ involvement which is clearly challenging.

Furthermore, rather uniquely, our patient had no respiratory symptoms and chest imaging did not reveal hilar lymphadenopathy. It was the PET-scan, performed as part of malignancy screen for resistant hypercalcaemia, which highlighted the diagnosis. However, due to lack of objective weakness on examination and normal CK the significance of this incidental nonspecific finding was deemed low.

A literature review has highlighted 12 similar cases where PET-scan, performed as malignancy screening for hypercalcaemia, incidentally, revealed intense, diffuse, and isolated muscular FDG uptake consistent with granulomatous myositis. CK was normal in all cases and no patients displayed the usual thoracic features of sarcoidosis. All patients were treated with high-dose steroids and achieved rapid, complete, and sustained remission.

**Case report - Key learning points:**

Sarcoidosis should be considered in patients presenting with symptomatic hypercalcaemia with no apparent causes and negative routine workup. The absence of proximal weakness or elevated muscle enzymes do not preclude the diagnosis of granulomatous myositis.

Severe hypercalcaemia revealing a diffuse granulomatous disorder limited to muscles might be life threatening and following literature review appears highly steroid-sensitive.

We describe a rare case where PET-scan incidentally revealed sarcoid-like myositis features which were subsequently demonstrated by muscle biopsy. Rheumatologists and physicians should be aware of this potential cause of markedly elevated calcium, even with normal CK and CXR and no prior sarcoidosis diagnosis. Earlier recognition of this disease phenotype can avoid unnecessary investigations, diagnosis delay and added stress to patients.

